# Multiwavelength Multipoint Observations of the October 28, 2021 Type III Radio Burst

**DOI:** 10.1007/s11207-026-02647-9

**Published:** 2026-04-13

**Authors:** Juan Carlos Martinez Oliveros, Vratislav Krupar, Tamar Ervin, Säm Krucker, Marc Pulupa, Samuel T. Badman, Juan Camilo Buitrago Casas, Surajit Mondal, Stuart D. Bale

**Affiliations:** 1 Space Sciences Laboratory, University of California Berkeley, 7 Gauss Way, Berkeley, CA 94720 USA; 2 Observatorio Astronómico Nacional, Universidad Nacional de Colombia, Bogotá, Colombia; 3 Goddard Planetary Heliophysics Institute, University of Maryland, Baltimore County, Baltimore, MD 21250 USA; 4 Heliospheric Physics Laboratory, Heliophysics Division, NASA Goddard Space Flight Center, Greenbelt, MD 20771 USA; 5 Center for Astrophysics, Harvard & Smithsonian, Cambridge, MA USA; 6 Center for Solar Terrestrial Research, New Jersey Institute of Technology, Newark, NJ 07102 USA; 7 Department of Physics, University of California Berkeley, 366 Physics North, Berkeley, CA 94720 USA; 8 University of Applied Sciences and Arts Northwestern Switzerland (FHNW), Bahnhofstrasse 6, 5210 Windisch, Switzerland

**Keywords:** Flares, Solar radio emissions, Type III, Magnetic fields, Corona

## Abstract

Type III solar radio bursts trace electron beams escaping from flares onto interplanetary magnetic fields, yet unambiguous source identification and continuous beam tracking remain challenging. We analyze a Type III event associated with a compact flare in NOAA AR 12887 studied with an unusually complete set of observables: X-ray imaging of the flare site, radio spectro-polarimetry with direction-finding, and multi-point in-situ particle and wave measurements. This synergy delivered two key results. (1) We identify the burst’s solar source region by combining the timing consistency between the flare evolution and the radio onset (after accounting for light-travel time) with the sense and degree of circular polarization. The polarization is consistent with emission on topologically open (or quasi-open) field rooted in a compact EUV arcade, as expected for outward-propagating o/x-mode radiation in a diverging flux system. (2) We localize the electron beam and follow its spatio-temporal evolution into the heliosphere by triangulating radio directions and correlating them with time-of-flight signatures in the in-situ electron data. The accompanying Langmuir-wave measurements constrain the characteristic cross-section of the guiding flux tube via the spatial coherence and bandwidth of the wave packets, providing an empirical estimate of the beam’s aperture. The magnetic context of AR 12887 shows a complex photospheric field with adjacent open corridors. This configuration could explain the rapid magnetic connectivity between a compact EUV arcade and interplanetary space, and clarifies why strong polarization can arise even when closed loops are present nearby. Together, these observations establish an end-to-end linkage from flare energy release to heliospheric propagation and provide a template for future coordinated studies that require coincident timing, imaging, polarization, radio direction-finding, and in-situ diagnostics to resolve electron escape pathways.

## Introduction

Solar flares and coronal mass ejections (CMEs) are the most powerful eruptive events in the solar system and the primary drivers of geoeffective space weather. These phenomena result from the large-scale restructuring of the solar coronal magnetic field. This restructuring, known as magnetic reconnection, abruptly releases and converts stored magnetic energy, manifesting in several primary ways: the intense heating of plasma, the dynamic ejection of coronal mass, and the acceleration of particle streams.

These physical processes generate a broad spectrum of observable radiation. The intensely heated plasma, for instance, produces the characteristic soft and hard X-rays seen in a flare from coronal and chromospheric sources, while the accelerated particle streams generate radio emissions (like Type III bursts) as they travel from the corona in and out into the chromosphere and interplanetary medium. Each observed frequency range is associated with a specific emission mechanism, giving direct clues into the physical conditions of the solar atmosphere or interplanetary space where that emission originates.

Historically, solar radio bursts have been classified into types based on their distinct morphological signatures in the radio dynamic spectrum. The temporal evolution of these features is a powerful diagnostic tool, as the emission frequency and drift rate directly signify the physical processes at work (Bastian, Benz, and Gary 1998). Type II radio bursts, generally are associated with major flares and/or CMEs and show a characteristic slow drift from higher to lower frequencies as they trace the propagating shock wave, typically lasting 5 to 30 minutes. In contrast, Type III radio bursts are fast-drifting signatures in the dynamic spectra, recognized as the signatures of flare-accelerated electron beams, typically with energies of 5 to 30 keV (e.g., Kane 1981; Wang et al. 2023). Apparent exciter speeds inferred from interplanetary Type III drift rates span a wide range and can be below $0.1c$ at low frequencies, depending on assumptions about the emission (fundamental versus harmonic) and transport/propagation effects. For example, statistical analyses of isolated interplanetary Type III bursts report inferred exciter speeds ranging from ∼ 0.02$c$ up to ∼ 0.35$c$ under different assumptions. Their characteristic fast frequency drift occurs because the emission is generated near the local plasma frequency, $f_{pe}$, (and its harmonics), which decreases as the beam propagates in the interplanetary medium. This emission process involves the propagating electrons generating a bump-on-tail distribution, which drives the nonlinear growth of Langmuir waves via the two-stream instability; these waves are then converted to transverse electromagnetic waves (e.g.,. Weber 1978; Krupar et al. 2015).

The association between large-scale Solar Eruptive Events (SEEs, such as solar flares and CMEs, and in-situ particle fluctuations is well-established. Early studies showed that SEEs are often accompanied by in-situ measurements of energetic electrons (van Allen and Krimigis 1965; Krucker et al. 1999). More recently, Wang et al. (2021) demonstrated a clear dependence of Type III burst properties on the characteristics of the associated SEEs, such as the electron spectral index. This connection highlights the role of Type IIIs as a diagnostic for particle acceleration. Spectroscopic radio observations in GHz have pinpointed the origin of many Type III bursts to electron beams generated in the core regions of coronal jets (Chen et al. 2013, 2018). These studies, such as Chen et al. (2018), trace the propagation trajectory of the Type III-emitting beams, suggesting they diverge from a reconnection null point within the jet structure before propagating into interplanetary space.

Connecting source region properties in the low solar atmosphere with an associated type III radio emission allows us to better understand solar eruption dynamics and their space weather consequences. This includes the connection between how energetic and complex an eruption is to the size of the region in the solar wind it disrupts or affects. Such connections require multi-messenger and multi-point observations.

On October 28, 2021 a strong X1 solar flare (SOL2021-10-28T1517 X1.0) occurred near the solar disk center and subsequently produced a coronal mass ejection and copious type III radio emission. Not only was this event one of the strongest observed to date during the rising phase of solar cycle 25, but its flaring signatures and type III radio emission were also measurable by four independent spacecraft (Wind, STEREO A, Parker Solar Probe and Solar Orbiter) spread over a range of heliocentric distances from 0.6- – 1 au and spanning a 60 degree wedge of longitude aligned well with the source active region.

The analysis techniques and data synthesis presented in this manuscript showcase a unique methodology to study powerful events, such as the SOL2021-10-28 X1.0 flare. This methodology, along with multi-point, multi-messenger datasets, allowed us to explore the causal and spatial relation between the erupting region and the energetic electrons it injected into interplanetary space. This approach provided two key results: (1) unambiguous source identification, using timing consistency at the cadence of the available radio and X-ray diagnostics; and (2) the localization and spatio-temporal evolution of the electron beam in the interplanetary medium, using radio direction-finding and in-situ electron detection. The assembled observations drew a clear through-line from a complex active region to an extremely powerful electron injection event, which populated flux tubes across a 50-degree swathe of longitude and survived out to 1 AU.

## Observations and Instrumentation

On October 28, 2021, active region NOAA 12887 (located at ∼S22^∘^W05^∘^ as seen from Earth) hosted the large SOL2021-10-28 1517 X1.0 solar flare. According to the GOES event list, the flare onset occurred at ∼ 15:17 UT and the event reached its maximum at 15:35 UT. Prior to the flare, the active region NOAA 12887 showed a complex magnetic field configuration, classified under the Hale system as having both $\beta $ and $\gamma $ components (e.g., Hale et al. 1919). This flare was associated with an interplanetary radio burst observed by multiple spacecraft in the heliosphere. Various aspects of this event have already been widely analyzed (e.g., Wang et al. 2023; Klein et al. 2022).

**Figure 1:**
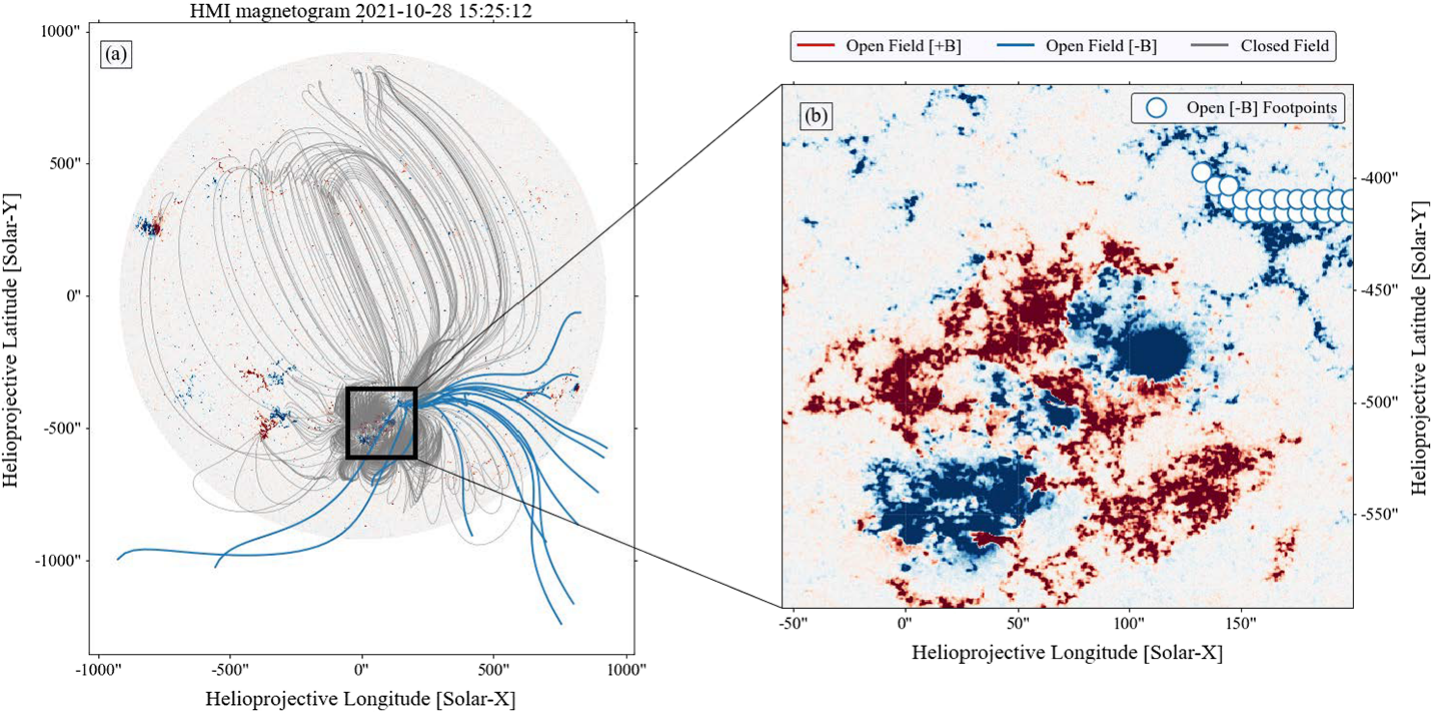
Panel (a): Global magnetic field distribution as modeled by the PFSS technique using the Python package sunkit-magex (formerly pfsspy, Stansby, Yeates, and Badman 2020). The model is derived from synchronic GONG data from October 28, 2021 14:00. Closed magnetic field lines are represented in gray, while open lines are in blue. The source surface height was fixed at 2.5 R_⊙_. Panel(b) highlights the photospheric locations of open field lines, denoted by the blue outlined circles. Only negative polarity open field regions are visible in this representation, with the densest concentration emerging from the unipolar region in the top right. The intricate magnetic configuration, featuring both $\beta $ and $\gamma $ components, within the region is highlighted. The area of interest is denoted by the box. This complex configuration is associated with the subsequent X1.0 flare that took place on 2021-10-28.

Figure 1 shows the topology of the magnetic field lines rooted in the active region as modeled by the Python package sunkit-magex (formerly pfsspy, Stansby, Yeates, and Badman 2020) which produces Potential Field Source Surface (PFSS; Altschuler and Newkirk 1969; Schatten, Wilcox, and Ness 1969) extrapolations. They are a computationally efficient method to predict the state of the global magnetic field but cannot capture dynamics, especially eruptive ones, or time dependent changes to the magnetic field configuration. PFSS extrapolations assume a magnetostatic (current-free) corona, and thus cannot produce any kinks or twists in magnetic field lines. There is an inner boundary condition (at 1 $R_{\odot}$) which is a photospheric field observation, and an outer boundary ($R_{ss}$) where the magnetic field is assumed to be purely radial. Within these boundary conditions, we can extrapolate the observed photospheric field to model the open and closed field line structure in the lower corona.

The PFSS model was calculated using synchronic GONG++ (Harvey et al. 1996) data from October 28, 2021 14:00. In Figure 1 panel (a), closed lines are drawn in gray and open magnetic field lines in blue. The source surface height for this analysis was set at 2.5 R_⊙_ (Hoeksema 1984). In panel (b), the blue circles show the photospheric locations where open field lines are present. The model only predicts negative polarity, open field lines in this region. This implies that the strong active region field is important to the global coronal topology and can connect to the solar wind.

### Type III Radio Burst

**Figure 2:**
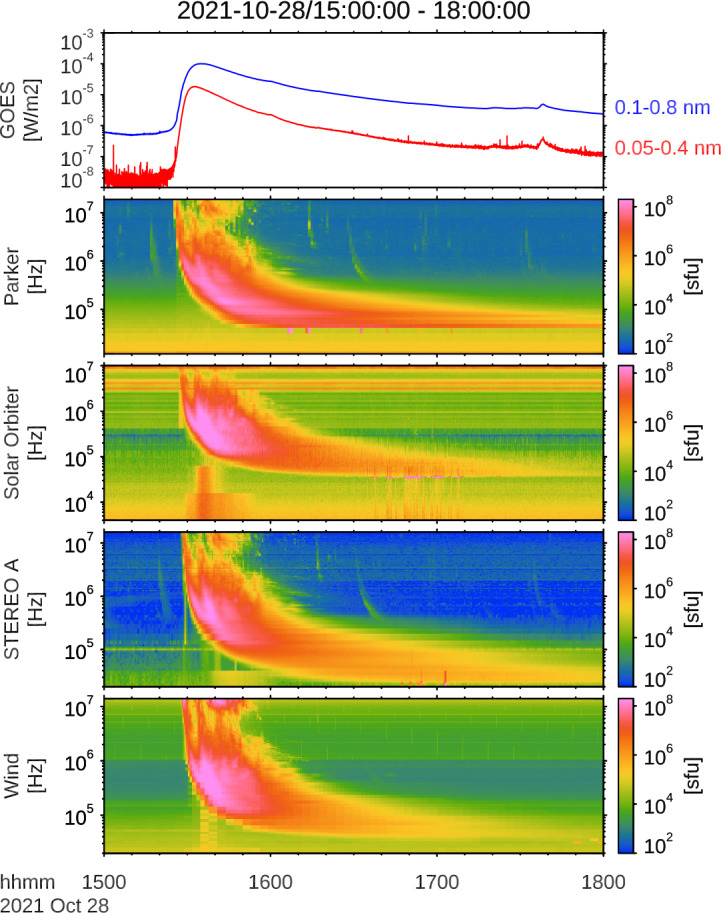
Dynamic spectra of the radio burst as detected by the STEREO A/WAVES, Wind/WAVES, Solar Orbiter/RPW and Parker Solar Probe/FIELDS instruments. The figure illustrates the typical Type-III radio burst characteristics, showing frequency and temporal structures at the onset and in higher frequency channels and that there are multiple injections contributing to the event. Langmuir waves are observed at and below the local plasma frequency in the Parker, Solar Orbiter and STEREO A data.

**Figure 3:**
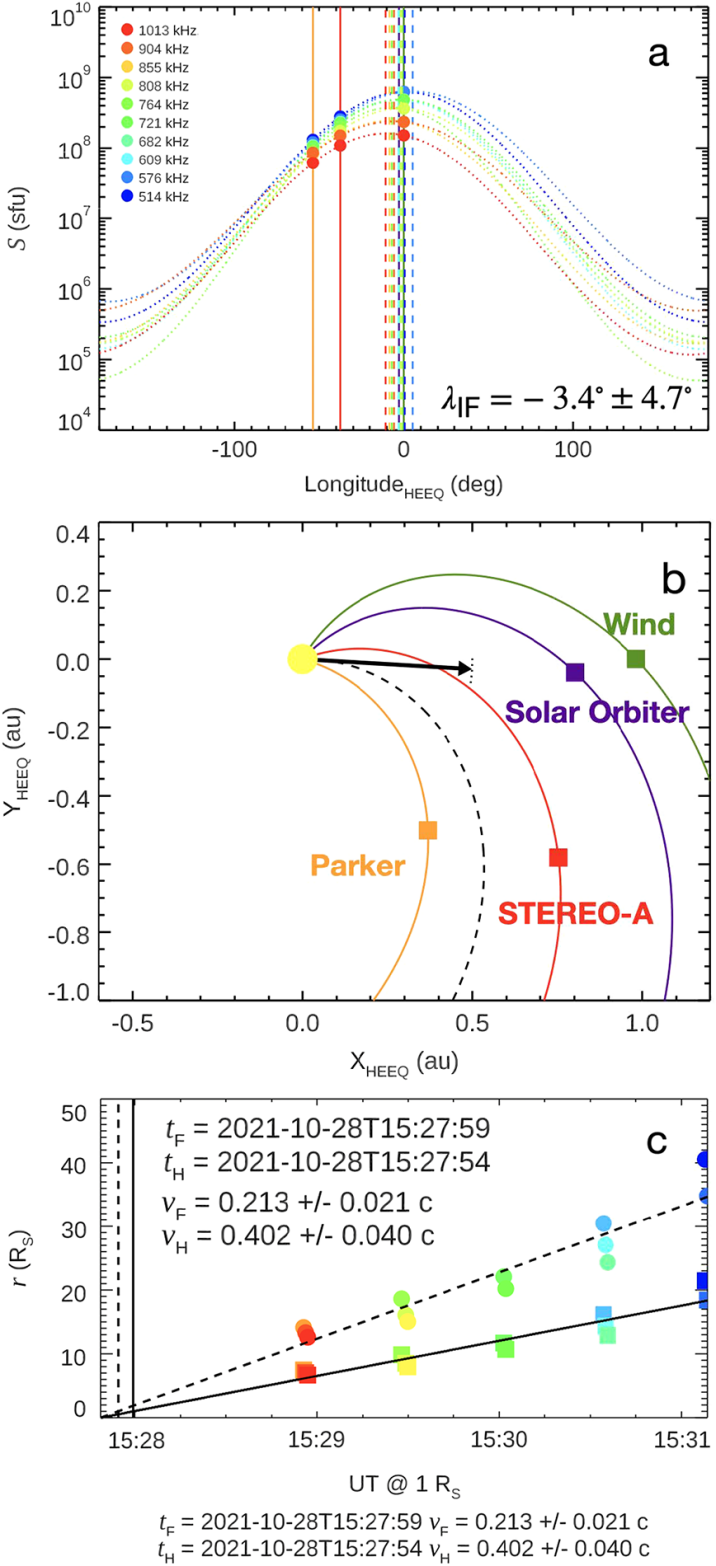
(a) Radio flux density $S$ versus spacecraft heliolongitude (HEEQ) for ten frequency channels between 514 and 1013 kHz (colors; see legend). Marker shape denotes spacecraft (PSP, Wind, STEREO-A). Solid vertical lines mark the spacecraft longitudes; the dashed vertical line marks the best-fit peak longitude $\lambda _{0}$; the dotted vertical line marks the flare longitude. (b) Spacecraft locations and nominal Parker-spiral connectivity at the event time; the arrow indicates the DF-inferred peak direction $\lambda _{0}=-3.4^{\circ}\pm 4.7^{\circ}$. The radio emission at 514 – 1013 kHz corresponds to $r\sim 7$ – $22~R_{\odot}$ (fund.) or $r\sim 14$ – $42~R_{\odot}$ (harm.); DF direction is at these heights. (c) Frequency-to-distance conversion using the Kruparova et al. (2023) density model and linear fits used to estimate exciter speeds under fundamental ($f=f_{pe}$) and harmonic ($f=2f_{pe}$) assumptions.

At the time of the flare event, four individual spacecraft were located within 1 AU on the earthward side of the Sun: STEREO-A (Kaiser et al. 2008), Wind (Wilson et al. 2021), Solar Orbiter (Müller et al. 2020a), and Parker Solar Probe (Fox et al. 2016). The spacecraft were spread across 60 degrees of Carrington longitude and spanned from 0.63 AU (PSP) to 1 AU (Wind and SolO) in radial extent (see Figure 3). All four spacecraft were equipped with radio spectrographs: STEREO-A/WAVES (Bougeret et al. 2008), Wind/WAVES (Bougeret et al. 1995), SolO/RPW (Maksimovic et al. 2021), and PSP/FIELDS/RFS (Bale et al. 2016). All four instruments detected a strong series of type III radio bursts, as depicted in Figure 2.

The respective onset times and spacecraft locations are detailed in Table 1. The timing is consistent with the flare onset, accounting for the radio emission travel time to each spacecraft. After correcting only for light-travel time, the associated Type III burst reaches its maximum flux near 1 MHz at approximately 15:36 UT in all spacecraft, close in time to the GOES soft X-ray maximum. We emphasize that this temporal proximity does not by itself imply a causal relationship.

**Table 1:** Type III Burst Radio Onset Times.

Spacecraft	Radial distance ($R_{\odot}$)	Radio onset (UT)
Parker Solar Probe	133.7	∼ 15:28:00
Solar Orbiter	172.8	∼ 15:28:30
Wind	213.2	∼ 15:29:00
STEREO-A	205.9	∼ 15:29:00

Because the onset times in Table 1 and the low-frequency spectrogram cadences are at the level of tens of seconds, we do not attempt sub-second timing correlations between flare-site hard X-ray spikes and the interplanetary radio onset in this event. The event shows a consistent morphology (i.e., its shape in the dynamic spectra) across all four spacecraft. It is composed of several successive electron injection events, which are visible as distinct features at high frequencies but merge together at lower frequencies. The event is extremely bright, with an unusually high radio flux surpassing $10^{8}$ sfu (Sasikumar Raja et al. 2022). The initial type III emission is also seen to be followed at high frequencies by a Type II radio burst, which was studied in detail by the community (e.g., Klein et al. 2022).

A notable difference is seen in the Parker Solar Probe data. The radio emission is observed to cut off sharply at a frequency of ∼ 40 kHz, while the other spacecraft at 1 AU (Wind, SolO, and STEREO-A) observe the burst smoothly down to the ∼ 20 kHz range. Because PSP was closer to the Sun (∼ 0.63 AU), it was embedded in a denser solar wind plasma. This denser plasma has a correspondingly higher local plasma frequency ($f_{pe}$). This indicates that the path from the electron beam to Parker eventually becomes opaque to radio transmission due to the density and plasma frequency falling with distance from the Sun. (see e.g., Badman et al. 2022).

**Figure 4:**
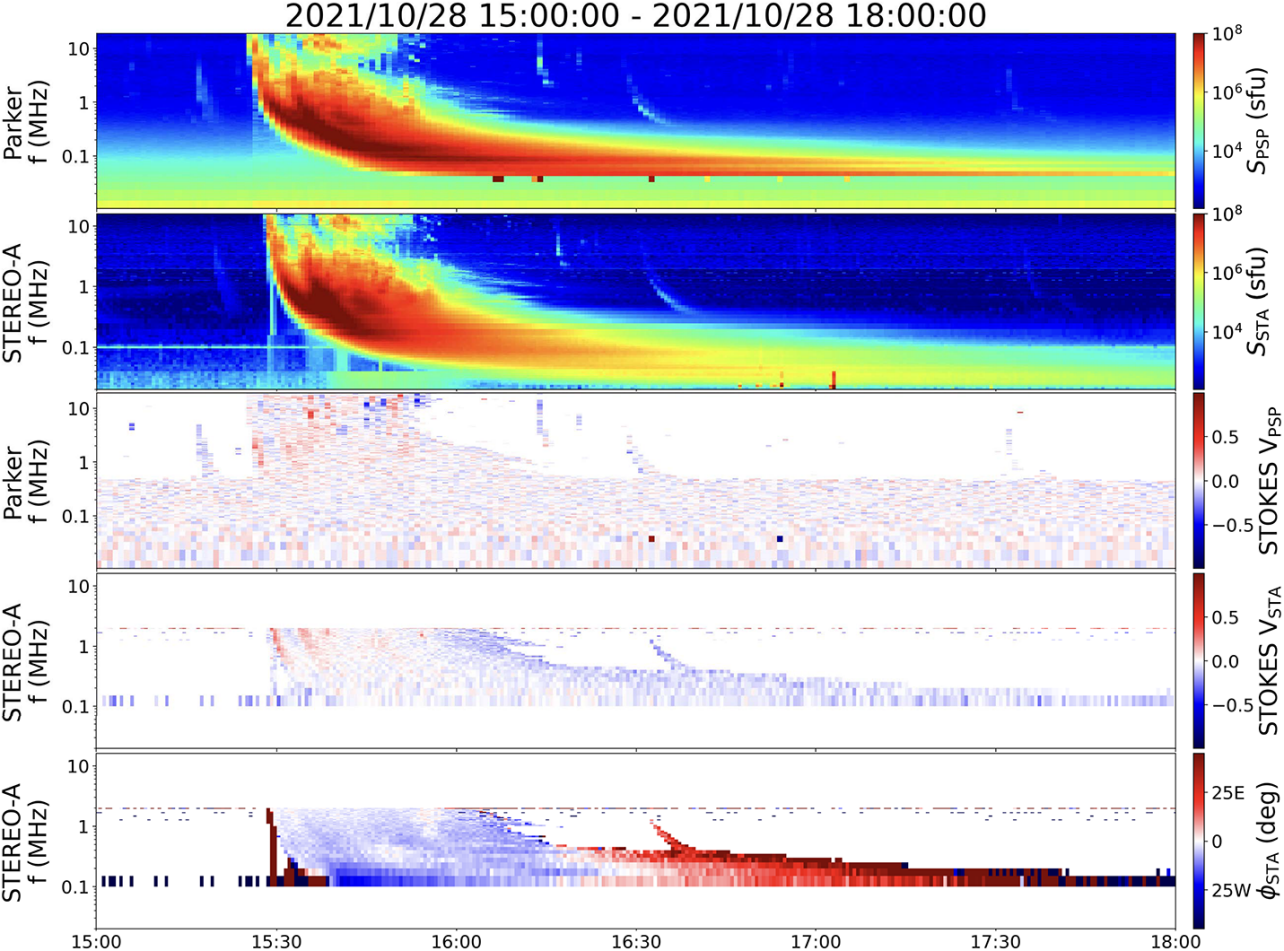
Circular polarization (Stokes V) of the radio emission observed by (a) Parker Solar Probe (PSP)/FIELDS and (b) STEREO-A/WAVES. The color scale in both panels represents the degree of circular polarization. Positive values (e.g., red) indicate Right-Hand Circular (RHC) polarization, while negative values (e.g., blue) indicate Left-Hand Circular (LHC) polarization. The main Type III bursts show a consistent positive (RHC) polarization in both datasets. The later Type II radio burst, visible in the STEREO-A data after 15:45 UT, is characterized by periods of alternating positive and negative polarization.

Lastly, Langmuir waves at and below the local plasma frequency are observed following the event in Parker, Solar Orbiter and STEREO-A, but not Wind (spurious brightnings at the plasma frequency, see Figure 2). The Langmuir waves occur as the event gets cutoff, so occur earlier at Parker and later at Solar Orbiter and even later at STEREO A, but not observed at all at Wind. Langmuir waves indicate the presence of an electron beam detected in situ at the spacecraft. The sequential observations are consistent with the electron beam traveling outwards from the Sun, and further show the injected electrons are spread along flux tubes at least 50 degrees apart (the angular separation between Parker and Solar Orbiter). The in-situ detection of Langmuir waves confirms that the electron beam reached the spacecraft and excited plasma waves near the local plasma frequency; however, this fact alone does not uniquely distinguish whether the observed electromagnetic emission was produced predominantly at the fundamental or the harmonic. Accordingly, we consider both assumptions in the drift analysis (Figure 3c) and use the observed polarization properties (Section 3; Figure 4) as an additional constraint on the likely dominant emission mode for the main burst.

From panel (b) in Figure 3 we see that nominal Parker spiral connectivity to the three spacecraft which see Langmuir waves are spread around the source longitude (black arrow), and that the one spacecraft which does not see evidence of electron injection (Wind) is the least well Parker spiral aligned with the source. It is worth noting that Wind is only 3 degrees separated from Solar Orbiter, so the lack of an in situ electron beam implies a quite sharp localization to the bundle of interplanetary magnetic field into which the type III burst travels, and suggests Wind and Solar Orbiter have quite different solar magnetic connectivity despite being relatively close in interplanetary space.

#### Direction Finding

Electron beams, such as those observed in type III radio bursts or shocks in type IIs, move away from the Sun, encountering decreasing local plasma densities as they propagate. This reduction in density subsequently lowers the frequency of the resultant radio emission, and therefore the information in the spectrograms (frequency vs time) can be inverted to assess the motion of electron beams. This enables independent verification that the injected electrons travel outwards, in the direction of the spacecraft constellation and lends further weight to the association with the X1.0 flare near disk center.

While all four spacecraft are equipped with direction-finding (DF) radio instruments, only STEREO-A and Wind currently offer calibrated DF data. Regrettably, the Wind spacecraft’s DF capabilities were compromised during this event due to the exceptionally high radio flux, making it challenging to retrieve reliable type III burst wave vector directions. To address this, we employed a novel technique developed by Krupar et al. (2024). This method localizes radio sources by fitting the von Mises distribution function to the radio flux measured by spacecraft with significant angular separation. However, the angular gap between the Solar Orbiter and Wind was minimal (around 3 degrees). Consequently, we applied this technique to data from Parker, STEREO-A, and Wind, excluding Solar Orbiter’s data due to electromagnetic interference (Maksimovic et al. 2021).

The method proceeds as follows. We assume the radio waves are beamed as a function of longitude in a von Mises or Gaussian circular distribution:

1 $$ S(\lambda )=\frac{S_{0}}{e^{\kappa}} e^{\kappa \cos (\lambda - \lambda _{0})} $$

Where $S_{0}$ is the peak radio flux, $\lambda _{0}$ is the longitudinal direction of this peak, and $\kappa $ denotes the distribution’s width, inversely proportional to $\sigma ^{-2}$ (a measure of the von Mises distribution’s dispersion). Using the known positions (i.e. longitudes) and radio brightness measured at each of the three spacecraft, we fit these distributions to determine the parameters. The fitted peak longitude $\lambda _{0}$ characterizes the direction of maximum observed radio intensity at the selected frequency and therefore constrains the beam direction at the heliocentric distance where emission at that frequency is generated (see Figure 3c), rather than the flare-site footpoint longitude.

Given that we had data from only three spacecraft and were fitting three parameters, we required a substantial number of frequency channels to validate our radio direction findings. We utilized 10 frequency channels observed by the three spacecraft, determining a direction of $S(\lambda )=-3.4^{\circ}\pm 4.7^{\circ}$, aligning with the central meridian where the solar flare was identified (Figure 3b). For the frequency range used in the direction-finding fit (514 – 1013 kHz), the density-model conversion in Figure 3c places the emission at heliocentric distances of approximately $r\approx 7$ – $22~R_{ \odot}$ (fundamental) or $r\approx 14$ – $42~R_{\odot}$ (harmonic), so the DF-derived longitude constrains the beam orientation in the inner heliosphere rather than the flare-site longitude. Figure 3a illustrates the measurements and fits of the radio flux density vs spacecraft longitude for these ten frequency channels.

Subsequently, we examined the radio frequency drift of the type III burst as observed by STEREO-A. We employed the density model by Kruparova et al. (2023), grounded in in situ observations by the Parker Solar Probe, to convert frequencies into radial distances from the Sun. We adjusted the observation times to align with the Sun’s surface for easier cross-referencing with other instruments. As previously mentioned, the emission was likely fundamental, resulting in an onset time of 15:29:59, consistent with other observations. Under the harmonic assumption, the inferred exciter speed is $v_{H}=0.402c$ (Figure 3c), corresponding to a kinetic energy of $\approx 47\text{ keV}$.

**Figure 5:**
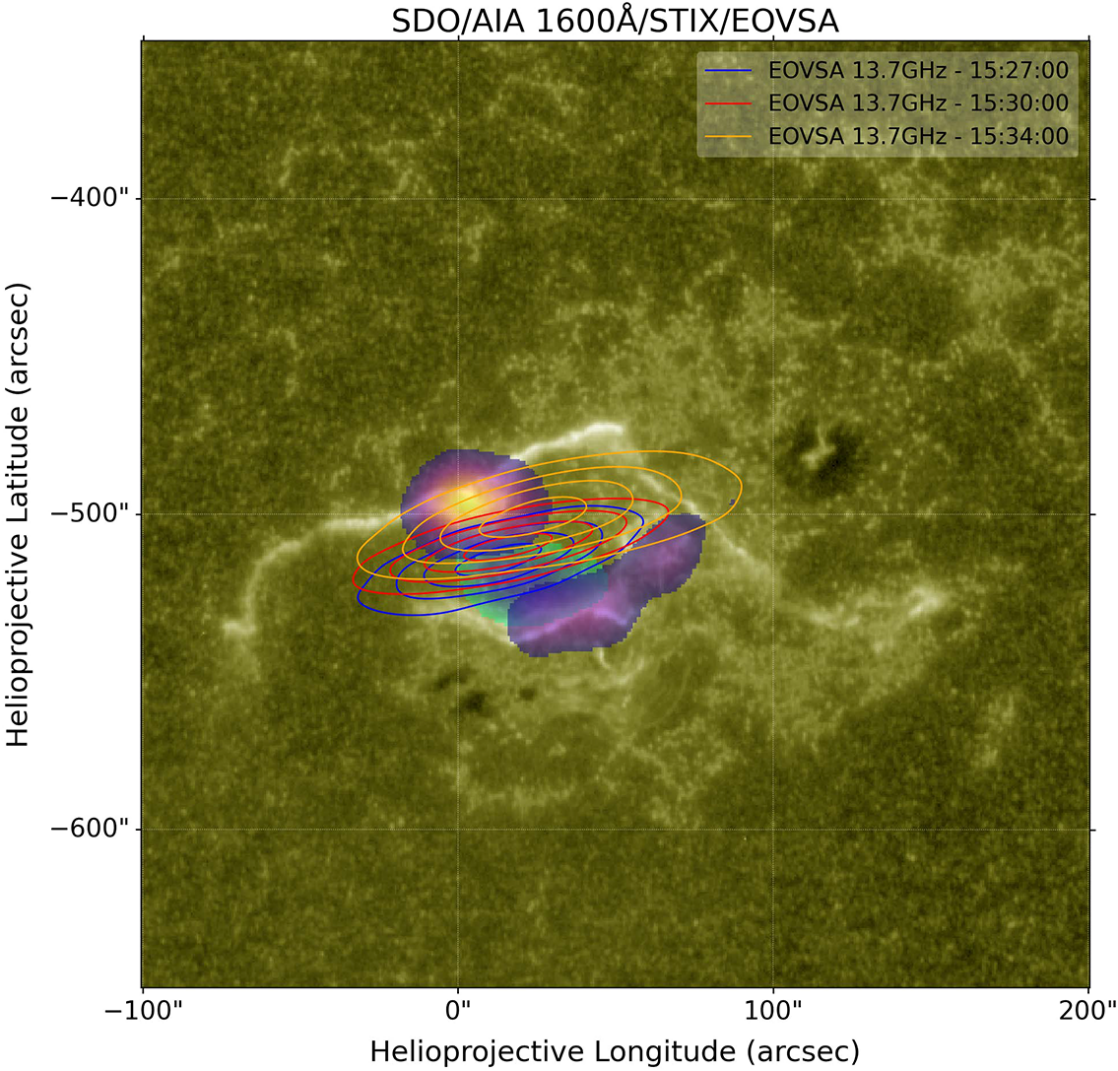
Multifaceted view of the SOL2021-10-28 solar flare (observations from 15:27-15:39 UTC). This figure combines X-ray observations, flare ribbons, and the dynamic evolution of radio sources to demonstrate the complexity of the flare. STIX X-ray sources are shown as color regions: 3-16 keV thermal emission (blue-cyan region) and 16-70 keV non-thermal emission (orange-yellow region). These are overlaid on an SDO/AIA 1600 Å image revealing the flare ribbons. EOVSA 13.7 GHz radio heliograph contours (blue, red, and orange lines) trace the temporal progression of the event at radio frequencies.

The drift-derived exciter speed depends on whether the emission is fundamental or harmonic. For the fundamental assumption we obtain $v_{F}=0.213c$ (Figure 3c), corresponding to a kinetic energy of $\approx 12\text{ keV}$, whereas for harmonic emission we obtain $v_{H}=0.402c$ ($\approx 47\text{ keV}$). The STIX non-thermal imaging shown in Figure 5 uses the 16 – 70 keV band, indicating that the flare-site hard X-rays sample the higher-energy portion of the accelerated distribution. In this framework, the Type III burst is driven primarily by the lower-energy (few-tens of keV) escaping beam, while the downward-going component produces the observed non-thermal hard X-rays. Subsequently, we examined the radio frequency drift of the Type III burst as observed by STEREO-A. We employed the density model by Kruparova et al. (2023), which is grounded in *in-situ* Parker Solar Probe observations, to convert the observed frequencies into radial distances from the Sun.

## Polarization

An additional aspect of the radio emission that can be examined with the STEREO A/WAVES instrument, and now at higher time resolution with Parker/FIELDS/RFS is the polarization signatures.

In examining the radio emission polarization behavior during the event, we found a strong agreement with the direction finding results. The radio emission observed by Parker Solar Probe (PSP) and STEREO-A show positive Stokes V, with a clearer signal in the STEREO-A data. The signature seems more apparent in the STEREO-A data which can be a result of the STEREO-A better time resolution at the time of the observation. Considering the direction findings, it makes sense that STEREO data has a stronger polarization signal than PSP since the emission is more toward STEREO-A. If we assume the emission is in the ordinary (o-) mode, the Right-Hand Circular (RHC) emission suggests magnetic field lines pointing toward the Sun, supporting our modeling results of negative-polarity field lines.

### Hard X-ray Emission

As of this publication, only one instrument can give us spatially resolved and spectroscopic observations in hard X-ray: the *STIX* instrument, onboard Solar Orbiter (Krucker et al. 2020; Müller et al. 2020b). The data from *STIX* can be easily compared with observations from other instruments such as the Solar Dynamics Observatory *HMI* and *AIA* instruments.

Figure 5 presents a multifaceted view of the SOL2021-10-28 flare, blending X-ray observations, UV flare ribbons, and the dynamic evolution of radio sources around 15:39 UTC. The base layer is an SDO/AIA 1600 Å intensity image, which reveals the flare ribbons. Overlaid on this are the STIX X-ray sources, shown as color regions: the non-thermal emission is represented by the orange-yellow region, while the thermal emission is shown by the blue-cyan region. This overlay shows the high-energy X-ray sources within the broader flare context. Further, contours in blue, red, and orange trace 13.7 GHZ radio snapshots from the Expanded Owens Valley Solar Array (EOVSA) radio heliograph at distinct flare stages (15:27, 15:30, and 15:34 UTC).

Specifically, the STIX data shows that the X-ray emission of the event comes from small localized regions. The EOVSA data shows the emitting region in radio grows more widespread as the source expands, but the centroid remains co-spatial and very well aligned both with the flare footpoints and the overall active region more broadly. These flare-site imaging observations locate the low-coronal acceleration/precipitation environment, while the interplanetary Type III direction-finding constrains the beam direction at radio-generation heights (tens of $R_{\odot}$). We connect these two diagnostics explicitly in Section 2.1.1.

### Linking the Flare-Site Hard X-rays to the Escaping Type III Beam

Magnetic reconnection is expected to accelerate electrons into both downward- and upward-propagating populations. The downward-directed component precipitates along closed flare loops and produces the observed non-thermal hard X-ray footpoint emission (STIX; Figure 5). The upward-directed component can access open (or quasi-open) field and escape into the heliosphere, where it drives Type III emission through beam-plasma instabilities.

The radio constraints analyzed here diagnose the escaping branch at the heights where the low-frequency emission is produced. In particular, our direction-finding analysis uses 514 – 1013 kHz, which correspond (under the adopted density conversion; Figure 3c) to heliocentric distances of order tens of solar radii. Therefore, the DF-derived peak longitude $\lambda _{0}$ should not be interpreted as the flare-footpoint longitude, but as the beam direction at the radio-generation heights.

The sense of circular polarization provides an additional constraint on the magnetic polarity and emission mode along the escape path. For the main Type III burst we observe positive Stokes $V$ (RHC) in both PSP and STEREO-A (Figure 4). Assuming ordinary-mode emission, the observed RHC sense implies field lines directed toward the Sun at the source, consistent with the negative-polarity open-field corridor inferred from the PFSS extrapolation (Figure 1). This supports the interpretation that the Type III-producing electrons escaped along negative-polarity open (or quasi-open) field rooted in AR 12887.

## Discussion

The observations and analyses presented in this article provide a consistent picture of a strong flare (SOL2021-10-28 1517 X1.0) inducing a very strong and widespread set of interplanetary type III radio bursts. The radio emission was detected by multiple spacecraft, including STEREO-A, Wind, Solar Orbiter, and Parker Solar Probe situated on and off the earth sun line at different distances. The unique constellation configuration enabled unambigous source association and a robust assessment of the spatial and temporal evolution of the type III emitting electron beams.

The event was preceded by a complex magnetic field configuration in the active region NOAA 12887, characterized by both $\beta $ and $\gamma $ components. The observations of Extreme UltraViolet (EUV) images and magnetograms reveal the intricate nature of the solar corona’s magnetic configuration, which likely contributed to the occurrence of the X1.0 flare.

The dynamic spectra of the Type III radio bursts revealed an extremely large peak radio flux as detected by all the radio instruments. (This high signal to noise enabled the direction finding analysis presented in Section 2.1.1). X1.0 flares have previously been associated with bright radio bursts but at $10^{8}$ sfu this event is an outlier. No remote observational evidence clearly explains this although we note the source active region was magnetically complex and the eruption was energetic enough to induce observable EUV waves and a CME (e.g., Wang et al. 2023; Klein et al. 2022)

The newly developed direction-finding technique by Krupar et al. (2024) was applied to determine the propagation direction of the radio waves in the interplanetary medium, indicating that the radio emission originated from regions close to the Sun – Earth direction, consistent with the X1.0 source and the locations in interplanetary space where in situ evidence of electron beams were observed. This in situ evidence took the form of Langmuir waves observed at 3 out of the 4 spacecraft. These waves suggested the event injected electrons onto flux tubes across 50 degrees of longitude, and the injection region was sharply cutoff such that beams arrived at Solar Orbiter but not Wind despite only being 3 degrees apart. This sharp edge highlights the importance of better modeling active region and interplanetary magnetic connectivity for the purposes of space weather predictions.

Hard X-ray emissions were studied using the STIX instrument onboard the Solar Orbiter. These observations were compared with data from the Solar Dynamics Observatory’s HMI and AIA instruments. The spatially resolved and spectroscopic observations of hard X-rays revealed a relatively compact source well-aligned with AIA 1600 and 1700 Å kernels. This alignment suggests a strong connection between the hard X-ray source and the underlying magnetic structures. EOVSA radio observations added to this picture that radio emitting electrons were generated in the low corona prior to their detection at interplanetary frequencies (Wang et al. 2023).

In conclusion, the comprehensive observations and analysis presented in this article provide a multi-faceted view of the interplay between solar flares, radio emissions, and hard X-ray emissions for energetic events. This case study illustrates how complementary measurements on and off the Earth-Sun line allow us to characterize the spatial and temporal evolution and extent of the electron beams that large flares can inject onto interplanetary field lines. Tying together the complex magnetic field configurations, radio propagation characteristics, and emission source locations requires the multi-point, multi-messenger measurements that define the current era of heliophysics. This observational capability will only become more critical heading into the solar maximum, as more frequent energetic events will provide the necessary data to constrain our models and improve space weather forecasting.

## Data Availability

Data from different spacecraft were analazied.
